# Modulatory Influence of Segmented Filamentous Bacteria on Transcriptomic Response of Gnotobiotic Mice Exposed to TCDD

**DOI:** 10.3389/fmicb.2017.01708

**Published:** 2017-09-07

**Authors:** Robert D. Stedtfeld, Benli Chai, Robert B. Crawford, Tiffany M. Stedtfeld, Maggie R. Williams, Shao Xiangwen, Tomomi Kuwahara, James R. Cole, Norbert E. Kaminski, James M. Tiedje, Syed A. Hashsham

**Affiliations:** ^1^Department of Civil and Environmental Engineering, East Lansing MI, United States; ^2^Center for Microbial Ecology, Michigan State University, East Lansing MI, United States; ^3^Institute for Integrative Toxicology, Michigan State University, East Lansing MI, United States; ^4^Department of Pharmacology and Toxicology, Michigan State University, East Lansing MI, United States; ^5^Department of Molecular Bacteriology, Institute of Health Biosciences, University of Tokushima Graduate School Tokushima, Japan

**Keywords:** TCDD, segmented filamentous bacteria, gnotobiotic mice, regulatory T-cells, gut dysbiosis, host microbe response, *B. fragilis*

## Abstract

Environmental toxicants such as 2,3,7,8-tetrachlorodibenzo-*p*-dioxin (TCDD), an aryl hydrocarbon receptor (AhR), are known to induce host toxicity and structural shifts in the gut microbiota. Key bacterial populations with similar or opposing functional responses to AhR ligand exposure may potentially help regulate expression of genes associated with immune dysfunction. To examine this question and the mechanisms for AhR ligand-induced bacterial shifts, C57BL/6 gnotobiotic mice were colonized with and without segmented filamentous bacteria (SFB) – an immune activator. Mice were also colonized with polysaccharide A producing *Bacteroides fragilis* – an immune suppressor to serve as a commensal background. Following colonization, mice were administered TCDD (30 μg/kg) every 4 days for 28 days by oral gavage. Quantified with the nCounter^®^ mouse immunology panel, opposing responses in ileal gene expression (e.g., genes associated with T-cell differentiation via the class II major histocompatibility complex) as a result of TCDD dosing and SFB colonization were observed. Genes that responded to TCDD in the presence of SFB did not show a significant response in the absence of SFB, and vice versa. Regulatory T-cells examined in the mesenteric lymph-nodes, spleen, and blood were also less impacted by TCDD in mice colonized with SFB. TCDD-induced shifts in abundance of SFB and *B. fragilis* compared with previous studies in mice with a traditional gut microbiome. With regard to the mouse model colonized with individual populations, results indicate that TCDD-induced host response was significantly modulated by the presence of SFB in the gut microbiome, providing insight into therapeutic potential between AhR ligands and key commensals.

## Introduction

Dysbiosis of certain key immune modulating commensals can influence host disposition to disease and environmental exposure (Snedeker and Hay, 2012). Specific individual bacterial groups can also revive or influence differentiation of T-cells observed in germ free or antibiotic-treated mice (Faith et al., 2014; Sefik et al., 2015; Honda and Littman, 2016). For example, multiple immune activating strains isolated from mice and human stool including segmented filamentous bacteria (SFB) induce Th_17_ cells in animal studies (Gaboriau-Routhiau et al., 2009; Ivanov et al., 2009; Atarashi et al., 2015; Tan et al., 2016). Alternatively, bacteria such as *Bacteroides fragilis* promote the expression of T_reg_ cells through polysaccharide A (PSA) production (Troy and Kasper, 2010). Dysregulation of T_reg_/Th_17_ cells can lead to various disease outcomes (Hand and Belkaid, 2010; Ivanov and Littman, 2010). For example, *Helicobacter pylori* succeeds in colonizing the gut by causing changes in both T_reg_ and Th_17_ cells (Kao et al., 2010). Thus imbalanced levels of T-cells can influence protection against pathogens or autoimmune diseases (Fantini et al., 2007; Peck and Mellins, 2010).

Attention to environmental exposure such as dioxins and other persistent organic pollutants has increased due to possible contributions to autoimmune diseases (Hertz-Picciotto et al., 2008), among others such as developmental disorders (Lee et al., 2007), obesity (Ibrahim et al., 2011), and diabetes (Taylor et al., 2013). Mediated in part through the aryl hydrocarbon receptor (AhR), 2,3,7,8-tetrachlorodibenzo-*p*-dioxin (TCDD, a porotype for studying the bioactivity for AhR) and other dioxins promote multiple toxic health effects including immune suppression (Kerkvliet et al., 2002, 2009). Thus, AhR ligands are also critically linked to the balance of T_reg_/Th_17_ cells (Ho and Steinman, 2008; Mustafa et al., 2008; Quintana et al., 2008; Veldhoen et al., 2008; Chmill et al., 2010; Marshall and Kerkvliet, 2010; Veldhoen and Duarte, 2010; Zhang et al., 2010). For example, AhR ligands have been shown to abrogate inflammation caused by Crohn’s disease (Benson and Shepherd, 2011), which may in part be initially caused by commensal dysbiosis. While not completely understood, the pleiotropy type relationship between AhR modulating environmental toxicants, host, and gut commensals may potentially be employed to therapeutically modulate health.

Emerging studies also suggest that environmental toxicants such as TCDD (Lefever et al., 2016; Stedtfeld et al., 2017b) and other AhR ligands interact with gut commensals (Hubbard et al., 2015; Zhang et al., 2015; Murray et al., 2016). Previous murine studies with a traditional gut microbiome found that TCDD induced structural shifts in key bacterial populations; with increasing abundance of SFB (Bhaduri, 2015; Stedtfeld et al., 2017a) and decreasing abundance of *Bacteroidetes* in response to TCDD (Lefever et al., 2016). Known opposing T-cell host responses to SFB and exposure to TCDD suggest that expansion of SFB could potentially abrogate or lessen TCDD-induced toxicity and differentiation of regulatory T-cells (Marshall et al., 2008; Ivanov et al., 2009). It was also unknown if SFB response was due to structural shifts in other bacterial populations (e.g., decreased abundance in *Bacteroidetes*), or due to TCDD-induced shifts in the host.

To answer these questions and examine the modulatory potential of an immune activating bacteria in animals exposed to TCDD, gnotobiotic mice were colonized with and without SFB. Mice were also colonized with immune suppressing (Round and Mazmanian, 2010; Troy and Kasper, 2010; Atarashi et al., 2011) PSA producing *B. fragilis* to serve as a commensal background. A separate group of mice was also mono-colonized with SFB or non-colonized to further verify the modulatory potential.

## Materials and Methods

### Animal Models and Bacterial Cocktails

Germ-free female C57BL/6 mice were bred and maintained at the Germ-Free Mouse Facility housed in the Unit for Laboratory Animal Medicine at the University of Michigan (Ann Arbor, MI, United States) and maintained in germ-free isolators. Mice were orally colonized with bacteria 4–6 weeks after birth (Supplementary Figure S1). TCDD dosing started 4 weeks after colonization. A previously described TCDD dosing regimen of 30 μg/kg (AccuStandard, New Haven, CT, United States) by oral gavage once every 4 days for 28 days (Fader et al., 2015; Nault et al., 2015) was used. Mice were dosed by oral gavage with 0.1 ml of sesame oil vehicle control (Sigma–Aldrich, St. Louis, MO, United States) or TCDD in sesame oil vehicle.

Results shown in this study are based on the following two experiments and animal numbers: Experiment 1 consisted of untreated (vehicle) with *B. fragilis* mono-colonization (*n* = 4), TCDD treated with *B. fragilis* mono-colonization (*n* = 4), an untreated (vehicle) with co-colonization of SFB and *B. fragilis* groups (*n* = 4), and TCDD-treated with co-colonization of both groups (*n* = 4).

To further verify modulation potential of SFB, experiments were replicated in the absence of *B. fragilis* including untreated (vehicle) uncolonized (UC; *n* = 4), TCDD-treated UC (*n* = 4), untreated (vehicle) with SFB mono-colonization (*n* = 4), and TCDD-treated with SFB mono-colonization (*n* = 4). One untreated mouse mono-colonized with SFB and one co-colonized treated mouse died prior to sacrifice. Mice had access to sterile water and food *ad libitum*. All animals received humane care in compliance with the animal use protocol approved by the University of Michigan, U of M Animal Welfare Assurance (A3114-01). Handling of blood and tissue exposed to TCDD was carried out as per MSU’s Environmental Health and Safety approved protocols under AUF numbers 02/14-030-00.

*Bacteroides fragilis* (DSM 2151) used for colonization was grown in Brucella broth (AS-105, Anaerobe Systems, Morgan Hill, CA, United States). *Candidatus* Savagella SFB-mouse-Japan, isolated as described previously (Kuwahara et al., 2011), was used for SFB groups. SFB was provided through an MTA between MSU and Kagawa University, Japan (No. AGR2015-00006 Kagawa University) and used as per approved protocols for handling BSL2 organisms. Prior to the association of bacteria into germ-free mice, qPCR was used to estimate abundance of bacteria and confirmed by Sanger sequencing of the 16S rRNA gene to ensure correct bacterial species. qPCR reactions included 1 ng of DNA extracted from fecal pellets, 18 μl Master Mix, and 1 μl of 10 mM primer mix in a 20 μl reaction. PCR conditions were as previously described (Bouskra et al., 2008). Briefly, cycling consisted of an initial 95°C for 5 min, 40 cycles of 95°C for 55 s, 60°C for 55 s, and 72°C for 1.5 min. Amplicons from three biological replicates (used for oral gavage) were purified using the Qiagen PCR purification kit and sequenced using the 96-capillary electrophoretic ABI 3730xl platform. Sequenced samples showed 99% identity with published mouse SFB sequences in the NCBI database (Accession Numbers: CP008713.1, AP012209.1, and AP012202.1). *B. fragilis* colonized groups were also inoculated with additional commensals including *Faecalibacterium prausnitzii* DSM 17677, *Ruminococcus bromii* Strain VPI 6883, *R. obeum* DSM 25238, *Butyrivibrio fibrisolvens* DSM 3071, and *Eubacterium rectale* DSM 17629 to serve as a background; however, only *B. fragilis* and SFB colonized to detectable levels in matrices tested.

### Collection of Fecal Pellets and Isolation of Tissue

Fresh fecal pellets were collected and analyzed every 8 days to ensure colonization, no contamination of unwanted bacterial groups, and examine the influence of TCDD on bacterial abundance. Fecal pellets were placed in RNA/DNA stabilizer (Zymo Research Corp, Irvine, CA, United States) and stored in a -20°C freezer. Whole blood, spleen, mesenteric lymph nodes, and intestinal tissues were collected at the time of sacrifice. Mice were weighed prior to sacrifice. Two intestinal sections were removed and used for subsequent analysis, including the cecum and the ileum. For the ileum, a 0.25 cm segment proximal to the cecum was removed and stored for all animals. For the cecum, sections of content and tissue were both removed and placed in the same vial. Intestinal tissue samples were immediately placed on RNA stabilizer and stored at -80°C.

### Transcriptomic Response Analysis with qPCR and nCounter^®^

RNA was extracted from ileum and cecum of all mice to evaluate the transcriptome response of SFB and *B. fragilis* in response to TCDD and the response of host immune cells. In detail, the PureLink RNA Mini Kit with Trizol (12183018A, Ambion/Thermo Fisher Scientific, Waltham, MA, United States) was used to extract RNA from mouse tissue and content. Two additional steps were used to digest DNA including the DNase 1 (Invitrogen/Thermo Fisher Scientific, Waltham, MA, United States) and Turbo DNase I Kit (Life Technologies/Thermo Fisher Scientific, Waltham, MA, United States). After each step, isolated RNA was quantified using a Qubit (Life Technologies), and assessed for purity using the Nanodrop ND-1000 UV–Vis spectrophotometer (Nanodrop Products, Wilmington, DE, United States).

2,3,7,8-Tetrachlorodibenzo-*p*-dioxin-induced expression of both bacterial members was monitored in the ileum and cecum using qPCR. For mRNA analysis of bacteria, qPCR primers were designed from sequences available for the colonized organisms. Genes targeting SFB functions were selected based on potential interaction with host immunity (Pamp et al., 2012) including the putative hemolysin A producing gene (BAK56433.1), and the putative rubrerythrin producing gene (BAK56170.1). Primers targeting *B. fragilis* gene clusters *wcfQ* (CAH07088.1) responsible for biosynthesis of PSA (Troutman et al., 2014) were selected due to known influence in signaling naïve T-cells differentiation into T_reg_ cells (Troy and Kasper, 2010). Primers were designed as previously described (Stedtfeld et al., 2008). Briefly, qPCR primers were designed from sequences downloaded from NCBI using Primer Express (Applied Biosystems) default settings; with a maximum length of 150 bases, and a theoretical melting temperature of 59°C. NCBI BLAST analysis (Altschul et al., 1997) was used to check theoretical specificity of designed primers against the GenBank database (Supplementary Table S1). cDNA was synthesized via random primers as instructed in the High Capacity cDNA Reverse Transcription Kit (Thermo Fisher Scientific, Waltham, MA, United States). qPCR was performed using a custom SmartChip^TM^ (Wafergen Biosystems, Fremont, CA, United States) with species-specific 16S rRNA gene and functional gene primers (Supplementary Table S1). Briefly, Wafergen’s Custom SmartChip^TM^ array allows for 5,184 qPCR reactions with 100 nl volumes to be run in parallel. Sample/primers were dispensed into the SmartChip^TM^ using a Multi-sample Nano-dispenser (Wafergen Biosystems, Fremont, CA, United States). PCR cycling conditions and initial data processing were performed as previously described (Wang et al., 2014; Stedtfeld et al., 2016). Amplification reactions on the SmartChip^TM^ consisted of 1× LightCycler 480 SYBR^®^ Green I Master Mix (Roche Inc., United States), nuclease-free PCR-grade water, 50 ng/μl cDNA template per sample, and 0.5 μM of each forward and reverse primer. Thermal cycling included an initial denaturation at 95°C for 3 min, followed by 40 cycles of denaturation at 95°C for 30 s, and annealing at 60°C for 1 min. Each primer/sample combination was tested in triplicate. Genetic copies were estimated as previously described (Looft et al., 2012). Negative controls with no templates were also included on the array.

2,3,7,8-Tetrachlorodibenzo-*p*-dioxin-induced ileal expression of host maker genes related to immune function was quantified using the nCounter^®^ mouse immunology panel. In detail, 200 ng of digested RNA was submitted per sample using the nCounter^®^(NanoString Technologies, Seattle, WA, United States). NanoString’s nCounter^®^ mouse immunology panel contains probes targeting 547 immunology-related mouse genes (Reis et al., 2011).

### qPCR of gDNA from Fecal Pellets

Genomic DNA was also extracted from fecal pellets 18–20 days after colonization and 21 days after initial treatment (following the sixth dose of TCDD) using the PowerSoil Extraction Kit (MoBio). qPCR was performed using species-specific 16S rRNA gene primers (Supplementary Table S1). Assays were performed in parallel using the Wafergen SmartChip^TM^ with the same reaction conditions described above for cDNA; however, only 0.1 ng/μl of gDNA was used per sample. Results showed colonization of SFB and *B. fragilis* in the groups expected to contain these populations.

### Flow Cytometry of Whole Blood, Splenocytes, and Mesenteric Lymph Nodes

Regulatory T-cell responses were measured in mesenteric lymph nodes, blood, and spleen. Trunk blood was collected into heparin-treated tubes followed by red blood cell lysis using a standard ACK lysing protocol (BD Biosciences, San Jose, CA, United States). Whole blood leukocytes were washed and counted, 1–2 × 10^6^ leukocytes were stained with antibodies for analysis by flow cytometry. Spleens and mesenteric lymph nodes were isolated and made into single-cell suspensions by removing the capsule and connective tissue via mechanical disruption. Cells were then washed and 1–2 × 10^6^ lymphocytes stained with antibodies for analysis by flow cytometry. Subsequently, Fc receptors on peripheral blood, lymph node, and spleen-derived leukocytes were blocked with an anti-mouse CD16/CD32 (BD Biosciences, San Jose, CA, United States), and the cells were stained for extracellular proteins using the following antibodies (Biolegend, San Diego, CA, United States) CD3, CD4, CD25, and NK1.1. Samples were incubated for 20 min at 4°C, and washed twice with FACS buffer (1× HBSS containing 1% BSA and 0.1% sodium azide). Next, cells were fixed with Cytofix (BD Biosciences, San Jose, CA, United States) and resuspended in FACS buffer. For intercellular protein staining, cells that were previously fixed after surface staining were permeabilized with FoxP3 Perm Buffer (Biolegend, San Diego, CA, United States) by incubating the cells in the perm buffer for 10 min, followed by the addition of the antibodies (FoxP3 and IL-17F (Biolegend, San Diego, CA, United States) directly to the cells and incubated for an additional 30 min at room temperature. Cells were washed four times in the Perm Buffer and after the last wash cells were re-suspended in FACs buffer. Cells were analyzed using a FACSCanto II Flow Cytometer (BD Biosciences, San Jose, CA, United States) and the data analyzed with Flowjo software (ver. 8.8.7 Treestar Software, Ashland, OR, United States), with a gating strategy as shown in Supplementary Figure S2. The percentage of Th_17_ cells was near the detection limit in measured matrices, as the background ranged from 0.001 to 0.08% in whole blood samples for IL17 staining.

### Statistical Analysis and Host/Bacterial Functional Gene Annotation

qPCR was analyzed for absolute abundance based on mass of tissue used to extract DNA and extraction yield, or normalized for relative expression of 16S rRNA gene specific to SFB or *B. fragilis*.

For data obtained using the nCounter^®^, expression counts were normalized to the geometric mean of multiple housekeeping genes including Rpl19, Ppia, G6pdx, Tubb5, Alas1, Tbp Gusb, Hprt, Gapdh (all had %CV <50) with an algorithm developed by NanoString Technologies. Genes with a maximum normalized expression count of 10 or less among all colonized groups were removed. The Shapiro–Wilk test was used to evaluate normality of data. If necessary, the variance between compared groups was corrected with the Geisser–Greenhouse method. For comparison of more than two groups, one-way ANOVA followed by a multiple comparison Sidak test was performed. When normal distribution was satisfied, a Student’s *t-*test was used for comparing differences between two groups. Otherwise, the non-parametric Mann–Whitney test was used. Statistical analysis and some plots were generated using Prism (version 7 for Windows; GraphPad Software, San Diego, CA, United States), additional plots were rendered using Excel or Cytoscape v. 3.3.0 (Institute for Systems Biology, Seattle, WA, United States). Genes were deemed significantly different if *p* < 0.05.

Genes that responded to TCDD and colonization were analyzed for enriched functions using the database for annotation, visualization, and integrated discovery (DAVID) v6.8 (Huang et al., 2009a,b). Gene ontology-biological processes (GO-BPs) were used for clustering the enrichment analysis. Functional groups with an enrichment score (ES)≥1.3 were considered significantly enriched, representing the -log scale geometric mean *p* < 0.05.

## Results

### Transcriptomic Response of Ileal Immune Cells

**FIGURE 1:**
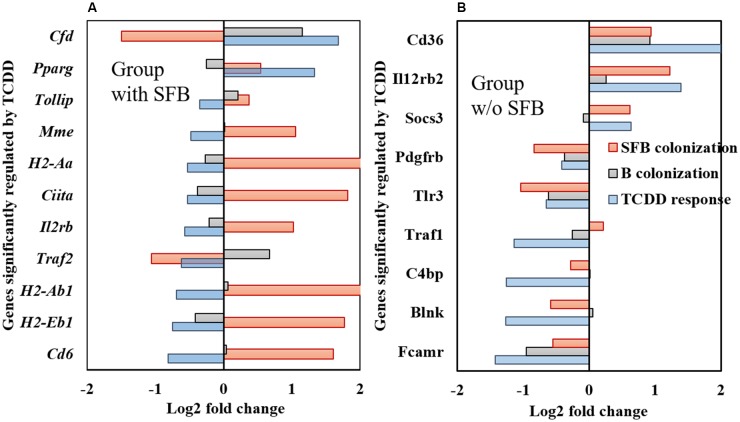
nCounter^®^ mouse immunology panel analysis of genes in ileal tissue that were significantly influenced in response to TCDD in mice **(A)** with SFB, and **(B)** without SFB. Line color indicates gene regulation due to TCDD (blue). For comparative analysis of genes significantly influenced by TCDD, fold change expression due to SFB and *B. fragilis* colonization is also shown regardless of significance. For comparative analysis of SFB colonization (red), the vehicle dosed group colonized with *B. fragilis* was compared with the vehicle dosed group co-colonized with SFB and *B. fragilis*. For comparative analysis of *B. fragilis* colonization (gray), the vehicle dosed group colonized with *B. fragilis* was compared with the uncolonized vehicle dosed group.

The influence of SFB on TCDD-induced transcriptomic response was measured using the nCounter^®^ mouse immunology panel (**Figure 1** and Supplementary Table S2). Comparisons were made between groups of mice (Supplementary Figure S1) that remained uncolonized (UC), mice mono-colonized with *B. fragilis* (B), mice mono-colonized with SFB (SFB), and mice co-colonized with both bacteria (SFB+B) to examine: (i) influence of TCDD in presence of SFB (SFB+B, **Figure 1A**) and (ii) influence of TCDD in absence of SFB (UC and *B. fragilis* groups, **Figure 1B**). The influence of SFB colonization was determined by comparing vehicle dosed SFB+B vs B groups; and the influence of *B. fragilis* was determined by comparing vehicle dosed UC vs B groups. Results showed that 11 genes were significantly influenced by TCDD in the presence of SFB (**Figure 1A**). Nine out of 11 of these genes had an opposing response between TCDD and SFB colonization, in terms of transcript regulation. The two genes with similar responses between SFB colonization and TCDD (*cfd*, *Il2rb*) had a conflicting response between TCDD and colonization of *B. fragilis*. The influence of SFB on TCDD-induced host response was further verified in comparing UC with SFB mono-colonized groups (Supplementary Figure S3).

Nine genes were influenced by TCDD in the absence of SFB (**Figure 1B**), all of which were no longer significantly up/down regulated in the presence of SFB. All nine of the genes influenced by TCDD in the absence of SFB were up/down regulated in the same direction as SFB colonization. Influence of SFB colonization in **Figure 1B** was measured by comparing SFB vs UC groups. TCDD-induced responses in groups with and without SFB exhibited opposing modulation of genes associated with immune cell function.

A majority of genes that responded to TCDD in presence/absence of SFB are related to T-cell differentiation including: *Il1β*, *Ciita*, *H2-Eb1*, and *H2-Aa* which were significantly upregulated in response to SFB, and were downregulated in response to TCDD. These genes encode the class II, major histocompatibility complex (MHC II), transactivator, which is required for SFB induction of Th_17_ cells (Lecuyer et al., 2012; Goto et al., 2014). The *Il1β* gene, which has also been shown to substitute TGFβ in differentiation of T-cells (Ghoreschi et al., 2010), was also upregulated with SFB and downregulated with TCDD (Supplementary Figure S3). In the group without SFB, TCDD induced downregulation of genes related to T-cells (*Traf1*) and activation of NF-κB (*Tlr3*) and genes related to regulation of IgA and IgM (*Fcamr*, *Pdcd1*) homeostasis (Kawamoto et al., 2012; Ouchida et al., 2012).

**FIGURE 2:**
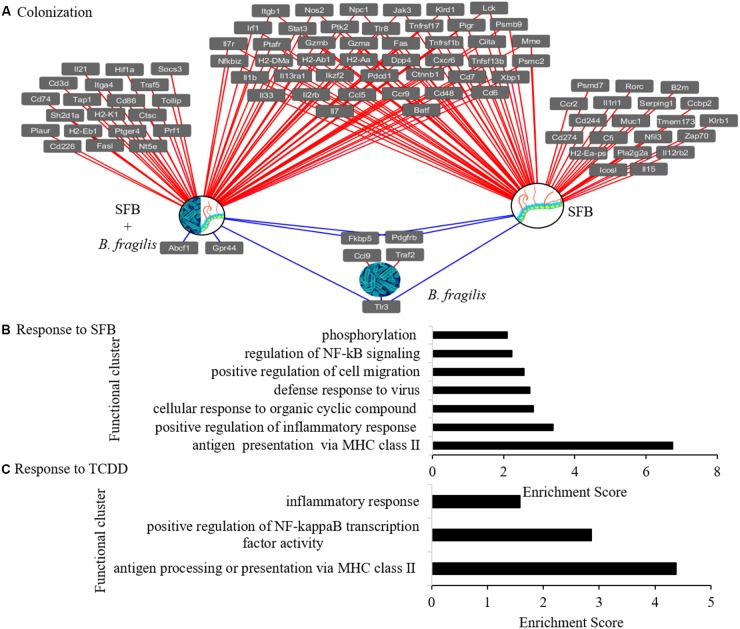
nCounter^®^ mouse immunology panel analysis and functional response. **(A)** Genes in ileal tissue that responded to SFB colonization. Line color and direction indicate upregulated (red) and downregulated (blue) genes. Abbreviations include mice that were mono-colonized with SFB (SFB), mono-colonized with *B. fragilis* (B) and co-colonized (SFB+B). **(B,C)** Functional clusters identified using the DAVID using nCounter^®^ mouse immunology panel analysis with gene expression of ileal tissue showing **(B)** clusters of genes that responded to colonization with SFB, and **(C)** clusters of genes that responded to TCDD in presence and absence of SFB. Functional clusters with scores =1.3 were included as significantly enriched.

Colonization with SFB had a greater overall influence on immune gene expression compared to TCDD (**Figures 1A,B, 2A**). In total, 93 genes were influenced by SFB colonization; and 83 of these were up-regulated. To identify the function of genes that responded to colonization and TCDD, the DAVID v6.8 (Huang et al., 2009a,b) was used. Seven and zero significant clusters were observed in response to SFB and *B. fragilis* colonization, respectively (**Figure 2B**). Three enriched clusters functionally associated with T-cell differentiation and inflammatory response (**Figure 2C**) were identified from the genes that responded to TCDD in one or more groups.

### Response of Regulatory T Cells

**FIGURE 3:**
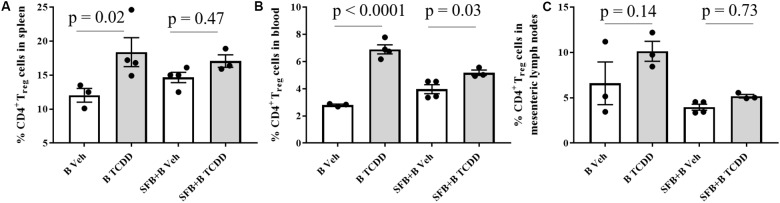
Percent CD4^+^ T_reg_ measured in **(A)** spleen, **(B)** whole blood, and **(C)** mesenteric lymph nodes in C57BL/6 female mice after TCDD (30 μg/kg) or vehicle (sesame oil) treatment by oral gavage once every 4 days for 28 days. Gray bars are TCDD dosed and white bars are vehicle dosed mice. Values represent mean percent and error bars represent standard error in presence/absence of SFB. *P*-values are shown between vehicle- (Veh) and TCDD-treated groups.

2,3,7,8-Tetrachlorodibenzo-*p*-dioxin-induced responses to T_reg_ cells in the mesenteric lymph node, blood, and spleen were less impacted in the presence of SFB, which is known to promote differentiation of cytokines toward Th_17_ cells (**Figure 3**). TCDD-induced differentiation of T_reg_ was greater in the group mono-colonized with PSA producing *B. fragilis*. The percent of CD3^+^ and CD4^+^ in the spleen and mesenteric lymph nodes of groups with SFB was also less influenced by TCDD (**Table 1**). Colonization and TCDD also tended to influence Th_17_ cells in an expected manner; however, Th_17_ measurements were near the limit of detection (Supplementary Figure S4).

**Table 1:** Body weight and T-cell number (CD3^+^ and CD4^+^) in spleen, blood, and mesenteric lymph nodes (lymph) after TCDD (30 μg/kg) or vehicle (sesame oil) treatment of mice by oral gavage once every 4 days for 28 days.

Group	Mouse wt (mg)	%CD3^+^ lymph	%CD4^+^ lymph	%CD3^+^ spleen	%CD4^+^ spleen	%CD3^+^ blood	%CD4^+^ blood
UC Veh	28.3 ± 0.8	48.2 ± 3.2	27.3 ± 1.4	27.8 ± 1.3	14.8 ± 0.9	18.0 ± 1.7	10.5 ± 0.6
B Veh	27.1 ± 0.6	45.2 ± 9.5	18.7 ± 4.6	29.2 ± 1.0	17.0 ± 0.6	22.9 ± 5.6	11.5 ± 2.9
SFB+B Veh	28.5 ± 0.6	48.8 ± 3.9	25.7 ± 2.2	25.0 ± 2.3	12.5 ± 1.4	20.7 ± 1.8	9.6 ± 1.0
UC TCDD	25.3 ± 0.6^∗^	43.1 ± 3.6	20.4 + 2.1	19.9 ± 2.5^∗^	10.3 ± 1.2^∗^	20.8 ± 2.3	9.8 ± 1.0
B TCDD	24.5 ± 1.0^∗^	72.9 ± 1.5^∗^	34.6 ± 1.1^∗^	23.9 ± 1.2^∗^	13.7 ± 1.0^∗^	20.7 ± 0.9	10.0 ± 1.0
SFB+B TCDD	26.1 ± 0.8^∗^	49.1 ± 4.0	25.6 ± 1.9	24.3 ± 2.3	12.8 ± 1.2	25.0 ± 2.0	12.0 ± 1.0


*Numbers indicate mean and standard error. Abbreviations identify mice that remained uncolonized (UC) or were mono-colonized with B. fragilis (B) and co-colonized (SFB+B). Vehicle dosed mice (Veh) and TCDD dosed mice (TCDD)*. ^∗^*Significant difference between TCDD and vehicle-dosed groups (p <0.05)*.

### Response of Colonized Bacteria

**FIGURE 4:**
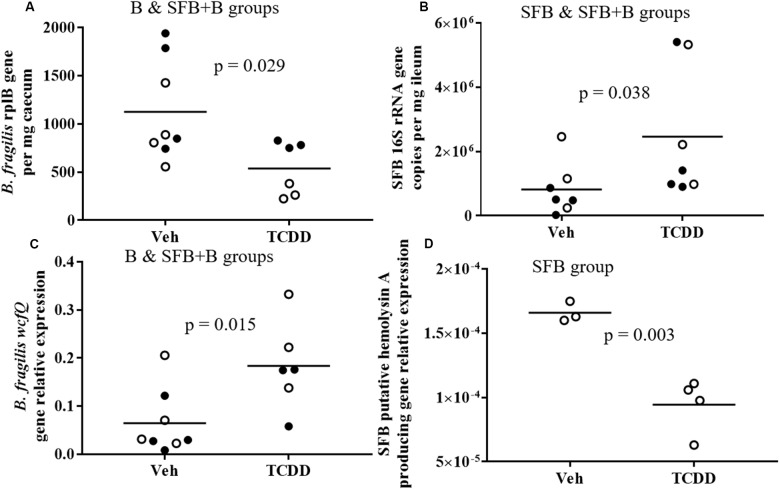
*Bacteroides fragilis* and SFB abundance and selected functional gene expression based on isolated RNA. **(A)** Absolute abundance of *B. fragilis* expression measured with specific *rplB* gene primers in cecum content from respective groups. **(B)** Absolute abundance of SFB expression using species-specific 16S rRNA gene primers from ileum of respective groups. **(C)** PSA biosynthesizing *wcfQ* gene normalized expression, measured with cecum content from respective groups. **(D)** SFB functional genes putative to interaction with host immunity (hemolysin A producing gene) normalized expression, measured with ileal RNA from SFB mono-colonized mice. SFB functional genes in co-colonized group are not included as the genes were expressed below detection limit in two or more biological replicates. Bars represent mean normalized abundance. Open dots indicate mono-colonized groups, closed dots indicate co-colonized groups. Abbreviations include vehicle dosed (Veh) and TCDD treated (TCDD).

qPCR analysis showed that TCDD significantly influenced the abundance of SFB and *B. fragilis* in the absence of other gut commensals (**Figure 4**). In detail, the absolute abundance of SFB was significantly higher in response to TCDD (**Figure 4B**). Increased SFB abundance was also verified with gDNA extracted from fecal pellets, and using assays targeting the SFB *fliC* functional gene (Supplementary Table S3). Overall, a 2.8-fold higher level of SFB (*p* = 0.038) was observed in response to TCDD. In contrast, the absolute abundance of *B. fragilis*, measured using species-specific *rplB* gene primers, significantly (*p* = 0.029) decreased 2.1-fold in mice dosed with TCDD (**Figure 4A**). The cecal and ileal abundance of *B. fragilis* and SFB did not differ among co-colonized or mono-colonization groups (Supplementary Table S3). Thus, bacterial abundances in both groups of mice were plotted and analyzed concurrently.

### Response of Select Functional Genes from Colonized Bacteria

The expression of select functional SFB and *B. fragilis* genes was also quantified via qPCR to examine responses putative to host interaction. In the ileum of the SFB colonized group, expression of the hemolysin A producing genes was downregulated in response to TCDD (**Figure 4D** and Supplementary Table S3). Hemolysin A has been shown to decrease the ability of host cells to phagocytize bacteria and undergo chemotaxis *in vitro* (Cavalieri and Snyder, 1982). Host macrophage recruiting genes were downregulated in the jejunum of mice dosed with TCDD in previously described studies (Fader et al., 2015). Down-regulation of this gene was only observed in the SFB mono-colonized group, as the expression was below the limit of detection in the group that also had *B. fragilis*.

Expression of the *wcf* gene involved in PSA production by *B. fragilis* was also measured. In detail, the *wcfQ* gene was significantly upregulated in the cecum of the *B. fragilis* colonized mice (**Figure 4C**) in response to TCDD. *B. fragilis* produce PSA as a control mechanism to induce regulatory T-cells and create a more self-tolerable growth environment (Round et al., 2011).

## Discussion

Comparative analysis between gnotobiotic mice colonized with and without SFB was performed to examine modulatory responses to TCDD. Noting the reductionist approach of the mouse model colonized with individual populations, the presence of SFB had an opposing response to TCDD in terms of host ileal immune gene expression, and helped modulate levels of TCDD-induced regulatory T-cells in blood, spleen, and mesenteric lymph nodes.

### Opposing Influence of SFB Colonization and TCDD on Host

Transcriptomic response fell into two categories including: (i) only responding to TCDD in opposition to SFB colonization and (ii) only responding to TCDD in the group without SFB. In the first category, genes influenced by TCDD would not have differed if had they not been expressed via colonization of SFB. This is evident in that none of the MHC II genes were differentially expressed in response to TCDD in groups without SFB. Within the second category, SFB reduced impact of TCDD on host immune gene expression. Verification of SFB influence in groups without *B. fragilis* further verifies opposing responses with TCDD (Supplementary Figure S3).

A majority of ileal immune genes were down-regulated with TCDD, with gene expression corresponding with differentiation of regulatory T-cells. In the presence of SFB, only two genes were significantly upregulated in response to TCDD (*Pparg* and *cfd*) in the group with background *B. fragilis*. The peroxisome proliferator-activated receptor-γ (*Pparg*) is a nuclear acceptor that inhibits NFκB activity. The downregulation of this gene has been associated with ulcerative colitis (Dubuquoy et al., 2003), and its increased expression correlates with anti-inflammatory responses, which is akin to what has been observed with TCDD (Benson and Shepherd, 2011). The genes that responded to TCDD in the absence of SFB (e.g., *Il12rb2*, *Cd36*) also regulate the number of regulatory T-cells and suppression of an inflammatory response (Zhao et al., 2008; Cecil et al., 2009).

### Influence of T-Cell Response in Health

2,3,7,8-Tetrachlorodibenzo-*p*-dioxin-induced response of regulatory T-cells in the mesenteric lymph nodes was less impacted in groups colonized with SFB, extending to blood and spleen matrices beyond the gut (**Figure 3**). Differences in regulatory T-cell responses among the colonized groups further indicate the influence of gut commensals on host disposition to TCDD. The impact of TCDD on regulatory T-cells has been shown to influence susceptibility to infection in the gut (Thigpen et al., 1975). However, the modulatory influence of SFB or Th_17_ inducers isolated from human stool (Tan et al., 2016) may help modulate susceptibility to bacterial infection and abrogate TCDD-induced response.

### TCDD-Induced Host Response Favors SFB

2,3,7,8-Tetrachlorodibenzo-*p*-dioxin-induced down-regulation of host immune genes that were activated with SFB colonization may provide a more favorable environment for SFB proliferation. This is evident in opposing functional inflammatory responses. Previous studies have found that mice deficient in mucosal immunity maintainers displayed a 10-fold increase in SFB abundance in fecal pellets (Upadhyay et al., 2012). Studies have also shown that mice lacking an enzyme critical to the production of IgA had an overgrowth of SFB despite the presence of other commensal organisms (Suzuki et al., 2004). TCDD also tended to reduce expression of the flagella receptor Toll-like receptor 5 gene *TLR5* gene 2.0-fold (*p* = 0.06) in the ileum of UC mice. Increased levels of flagella producing bacterial members have previously been observed in mice lacking the *TLR5* gene (Cullender et al., 2013). As such, TCDD appears to repress host immune cell gene expression in the gut, permitting proliferation of SFB. Downregulation of mRNA from measured SFB functional genes putative to evasion from host defense has similar implications.

A similar shift in SFB abundance was also observed in mice with a traditional gut microbiome; however, it was unknown if this was due to the expansion of other bacterial populations or changes in nutrients caused by commensal fluctuations. In detail, previous studies described an increased ratio of *Firmicutes* to *Bacteroidetes* in response to TCDD (Lefever et al., 2016; Stedtfeld et al., 2017a), and higher levels of *Proteobacteria* (Stedtfeld et al., 2017b). The expansion in these predominantly flagella producing groups (Lozupone et al., 2012) may in part influence the relative levels of *Bacteroidetes* and SFB. However, similar shifts in both bacteria in the gnotobiotic mice indicate this response can occur solely via TCDD-induced modulation of the host.

### TCDD-Induced Host Response Influences *B. fragilis*

The abundance of *B. fragilis* decreased in response to TCDD in the gnotobiotic mice. While the reduced abundance of *Bacteroidetes* was previously observed in TCDD-dosed mice with a traditional gut microbiome (Bhaduri, 2015; Lefever et al., 2016), the causation for this dysregulation is unclear. Previous studies performed by Round et al. (2011) suggest that *B. fragilis* favors an immunosuppressed environment, and that expression of *B. fragilis* PSA is a defense or colonization mechanism to increase differentiations of T_reg_ cells. In accordance, it would seem that PSA-producing genes should be downregulated to balance the levels of T_reg_ cells induced by TCDD. However, the upregulation of mRNA genes clusters related to the production of PSA indicates that *B. fragilis* are in a state of distress in response to TCDD. In addition, TCDD did not significantly influence the abundance of *Bacteroidetes* or other key commensals when tested *in vitro* in this (Supplementary Figure S5) or previously described studies (Lefever et al., 2016). Collectively, shifts in *B. fragilis* function and abundance also appear to be the result of TCDD-elicited changes in host.

### Relevance of TCDD Dose

Considering a half-life of 7–11 days for TCDD (Gasiewicz et al., 1983; Birnbaum, 1986), mice in this study had an estimated concentration of 60.5–90.7 μg/kg TCDD at sacrifice. One previously measured human exposure event resulted in comparable levels; with lipid-adjusted blood levels of 56 μg/kg TCDD in children near a 1976 plant accident in Seveso, Italy (Bertazzi et al., 1993). In addition, the lowest observable effect level of TCDD on inhibiting an immune response (IgM secretion) was lower in human (0.3 nM) than mice (3 nM) in lymphocytes tissue tested *in vitro* (Wood et al., 1993). We have also observed significantly higher levels of SFB at doses ≥1.0 μg/kg TCDD in other studies (Bhaduri, 2015). Thus, similar responses to those described here may occur with 10- to 100-fold less TCDD, warranting additional experiments. While environmental levels of TCDD are typically lower than that used in this study and are thought to be decreasing in the environment, cumulative exposure to other AhR ligands such as polyaromatic hydrocarbons and flame retardants is increasing (Lim et al., 2008; Wiseman et al., 2011).

### TCDD versus Other AhR Ligands

The AhR ligand response demonstrated in this study differs from those published by Zhang et al. (2015). In detail, their study with wild-type mice orally dosed with 2,3,7,8-tetrachlorodibenzofuran (TCDF), a less potent AhR activator, heightened host inflammation, reduced levels of SFB, and increased abundance of *Bacteroidetes* over *Firmicutes*. However, our results showed reduced levels of host immune gene expression, which has also been previously described in the jejunum in response to TCDD (Fader et al., 2015) and AhR activation (Monteleone et al., 2013). Dissimilar responses may be due to differences in AhR ligand potency, which has been reported in a study comparing TCDD and 6-formylindolo[3,2-b]carbazole (Farmahin et al., 2016). Future studies addressing AhR activity and influence to host and gut microbes in response to different ligands throughout the gut are necessary (Zhang et al., 2017).

### Differences in Murine and Human Models

Before inferring if TCDD and presence/absence of SFB would have a similar influence on humans compared to the murine model, both the host and microbial phenotype must be considered. Differences in host phenotype have been described previously (Kreisman and Cobb, 2011), with varied gene expression in response to TCDD depending on the measured tissue and host species (Kovalova et al., 2017). In regards to microbiota, recent meta-analysis has shown that 90 and 89% of bacterial phyla and genera, respectively, are shared between mice and the human gut microbiota (Krych et al., 2013), with the most abundant bacterial species being common in both. Studies examining the presence of SFB in human microbiome have conflicting results (Klaasen et al., 1993; Qin et al., 2010; Caselli et al., 2013; Yin et al., 2013; Shukla et al., 2015), which may be due to variability in the methods or matrices used for measuring SFB. However, immune-activating bacterial groups isolated from human gut microbiome appear to have a similar response to SFB in the capacity to differentiate T-cells in mice (Tan et al., 2016). Commercially available probiotic consortia have also been shown to contain Th_17_ cell inducers in mice (Tan et al., 2016), highlighting the therapeutic potential of immune-activating bacteria to modulate host response to TCDD.

Collectively, the presence of SFB significantly influenced host response to TCDD in the matrices measured. TCDD exposure also appeared to provide a more favorable environment for SFB. *B. fragilis* also responded to TCDD-induced shifts in the host, expressing higher levels of the *wcfQ* gene, a PSA-producing gene cluster. TCDD did not appear to influence abundance of these bacterial groups *in vitro* in TCDD-dosed batch reactors (Supplementary Figure S5), further suggesting that the bacterial response of SFB and *B. fragilis* is in part due to TCDD induced influences on the host.

Follow-up studies are underway to further elucidate causation of functional dysbiosis with *B. fragilis*. This will include the analysis of additional genes related to host interaction and immune tolerance. Further identification of key bacterial species and functional genes may facilitate attempts to manipulate the intestinal microbiome toward a protective and healthy state. Future studies will also examine the ability of other human bacterial commensals to influence TCDD-induced response in the absence of SFB. These studies may lead to new therapeutic approaches to treat intestinal pathogens and resulting autoimmune diseases.

## Author Contributions

RS, TS, and SH wrote the manuscript; RS, SH, JT, NK, RC, JC, and TK contributed to experimental design; and SH, BC, RC, TS, RS, SX, and MW aided in collection of tissue, measurements, and data analysis.

## Conflict of Interest Statement

The authors declare that the research was conducted in the absence of any commercial or financial relationships that could be construed as a potential conflict of interest.
