# Supplementary material for: Modulatory Influence of Segmented Filamentous Bacteria on Transcriptomic Response of Gnotobiotic Mice Exposed to TCDD
Source: Front Microbiol. 2017 Sep 7;8:1708. doi: 10.3389/fmicb.2017.01708 (PMC5594080; doi:10.3389/fmicb.2017.01708)
Supplement: Supplementary file 1 [file Data_Sheet_1.DOCX]

**Supplemental Material**

**Modulatory influence of segmented filamentous bacteria on transcriptomic response of** [**gnotobiotic**](http://www.ncbi.nlm.nih.gov/pmc/articles/PMC3918800/) **mice exposed to TCDD**

**Methods**

***Commensal response to TCDD in-vitro:*** Microcosm studies were performed to examine the commensal response to TCDD outside of the host. In detail, experiments were performed in sterile 8 ml vials in a 37 °C anaerobic chamber (with four space heaters and a staged Honeywell Temperature controller) under a 5% CO_2_-5% H_2_-90% N_2_ atmosphere. Prior to use, sterile media and vials were allowed to equilibrate in the anaerobic environment for 72 hours (this was also done to ensure sterility). One liter of bioreactor medium was prepared as previously described (Robinson et al., 2014). Five out of fifteen vials were spiked to have a final concentration of 30 nmol/l of TCDD and 0.87 μl/ml sesame oil, 5 were spiked with sesame oil controls, and 5 remained naive. Fecal pellets, stored in sterile cryogenic vials at −80 °C, were used to inoculate microcosms. Prior to inoculation, fecal pellets were re-suspended in sterile, anaerobic phosphate-buffered saline at a concentration of 25% (wt/vol), vortexed at maximum speed for 5 min, large particulates were removed by gravitational settling for 5 min, and the supernatants were used for inoculation of microcosms. Exactly 1.6 ml of 25% fecal slurry inoculum was inoculated into each test vial (8 ml volume) with a sterile needle and syringe, giving a final concentration of the fecal suspension of 1.63% (wt/vol). Post inoculation, microcosms were continuously mixed using a rotational shaker in an anaerobic chamber (Coy Laboratory Products Inc, Grass Lake, MI).

Microcosm samples (500 μl) were collected at time 0, 1, 2, 3, 6, 14 days post-inoculation using a sterile syringe and needle. Sample collected at day 0 were before addition of sesame oil and TCDD. Collected samples were immediately centrifuged at high speed for 15 min, supernatant was removed, RNA/DNA stabilizer was added, and samples were stored -80 °C until DNA extraction. DNA was extracted as described above and analyzed for 16S rRNA gene.

***Analysis of 16S rRNA genes from in-vitro microcosms:*** Amplicon sequencing libraries of the bacterial 16S V4 hypervariable region were made following the Schloss Lab protocol (Kozich et al., 2013). After PCR the reaction outputs were normalized using Invitrogen SequalPrep DNA Normalization Plates and then pooled. Completed library pool was QC'd and quantified using a combination of Qubit dsDNA HS, Caliper LabChipGX HS DNA, and Kapa Illumina Library Quantification qPCR assays. The pool was loaded on an Illumina MiSeq v2 standard flow cell and sequencing was performed in a 2x250 bp paired end format with a v2, 500 cycle reagent cartridge. Sequencing primers specific for the V4 region (515f/806r) were added to the appropriate wells of the reagent cartridge as described in (Kozich et al., 2013). Base calling was done by Illumina Real Time Analysis (RTA) v1.18.64 and output of RTA was demultiplexed and converted to FastQ format with Illumina Bcl2fastq v1.8.4. Sequences were analyzed using Qiime 1.

**Table S1.** qPCR primers used in this study to verify presence/absence in [gnotobiotic](http://www.ncbi.nlm.nih.gov/pmc/articles/PMC3918800/) mice and examine functional expression. Primer name refers to primer notation in original reference.

| Target | Primer name | Sequence |  | Reference |
| --- | --- | --- | --- | --- |
| Universal bacteria 16S rRNA gene | 340F  541F | ACTCCTACGGGAGGCAGCAGT  ATTACCGCGGCTGCTGGC |  | (Barman et al., 2008) |
| *Butyrivibrio fibrisolvens* H17c | ButFib2F  ButFib2R | ACCGCATAAGCGCACGGA  CGGGTCCATCTTGTACCGATAAAT |  | (Stevenson et al., 2007) |
| *Firmicutes* | Firm934F  Firm1060R | GGAGYATGTGGTTTAATTCGAAGCA  AGCTGACGACAACCATGCAC |  | (Guo et al., 2008) |
| *B. fragilis* | AllBac296F  AllBac412R | GAGAGGAAGGTCCCCCAC  CGCTACTTGGCTGGTTCAG |  | (Layton et al., 2006) |
| *Bacteroides* | BactF285  UniR338 | GGTTCTGAGAGGAGGGTCCC  GCTGCCTCCCGTAGGAGT |  | (Barman et al., 2008) |
| *Clostridiales* | UniF338  C.cocR491 | ACTCCTACGGGAGGCAGC  GCTTCTTAGTCAGGTACCGTCAT |  | (Barman et al., 2008) |
| *Clostridium* cluster IV | S-∗-Clos-0561-a-S-17  S-∗-Clept-1129-a-A-17 | TTACTGGGTGTAAAGGG  TAGAGTGCTCTTGCGTA |  | (Van Dyke and McCarthy, 2002) |
| *Clostridium* cluster IV | sg-Clept-F | GCACAGCAGTGGAG |  | (Matsuki et al., 2002) |
| (*Clostridium leptum* subgroup) | sg-Clept-R3 | CTTCCTCCGTTTTGTCAA |  | (Larsen et al., 2010) |
| *Clostridial cluster IV (Clep)* | Clep866mF  Clept1240mR | TTAACACAATAAGTWATCCACCTGG  ACCTTCCTCCGTTTTGTCAAC |  | (Lay et al., 2005; Sghir et al., 2000) |
| *Clostridium* cluster XIVa–XIVb | g-Ccoc-F | AAATGACGGTACCTGACTAA |  | (Matsuki et al., 2002) |
| (*Clostridium coccoides* subgroup) | g-Ccoc-R | CTTTGAGTTTCATTCTTGCGAA |  | (Larsen et al. 2010) |
| *Clostridium*  cluster XIVa | Erec482F | CGGTACYTGACTAAGAAGC |  | (Rinttilä et al., 2004) |
| (*Clostridium*  *coccoides–*  *Eubacterium*  *rectale* group) | Erec870R | AGTTTYATTCTTGCGAACG |  |  |
| cluster IV *Ruminococcus* spp. (brominii) | Rflbr730F  Clep866mR | GGCGGCYTRCTGGGCTTT  CCAGGTGGATWACTTATTGTGTTAA |  | (Walker et al., 2005) |
| *Roseburia spp.* & *E. rectale* | RrecF  Rrec630mR | GCGGTRCGGCAAGTCTGA  CCTCCGACACTCTAGTMCGAC |  | (Ramirez-Farias et al., 2009; Walker et al., 2005) |
| *Enterobacteriaceae* | Uni515F  Ent826R | GTGCCAGCAGCCGCGGTAA  GCCTCAAGGGCACAACCTCCAAG |  | (Barman et al., 2008; Lane, 1991) |
| but genes *Faecalibacterium prausnitzii* | G_Fprsn_F  G_Fprsn_R | GACAAGGGCCGTCAGGTCTA  GGACAGGCAGATRAAGCTCTTGC |  | (Vital et al., 2013) |
| SFB | SFB_736F  SFB_844R | GACGCTGAGGCATGAGAGCAT  GACGGCACGGATTGTTATTCA |  | (Bouskra et al., 2008) |
| *R. obeum* | ROB3, ROB2 | TGAGGAGACTGCCAGGGA  CTCCTTCTTTGCAGTTAGGT |  | (Wang et al., 1997) |
| SFB *fliC* gene |  | TGGGGATCGATGGTATGAAT  GCTCCAAGTTTAGTTCTTGCATT |  | (Prakash et al., 2011) |
| SFB hemolysinA gene |  | GAGAGAGTTGTAGTGTTTGAAAGGATGA  CAATTGATGCAAAATCCCCTTTAT |  | This study |
| SFB rubrerythrin gene |  | GGAGAAGTTTTTTATAAGGCTCAAGATG  CTGGAGCATATTCCCCATGTTC |  | This study |
| *B. fragilis wcfP* gene |  | GACTCTGATTGAAGCGCAAGCT  CCAAGTTGAAGGATCTCGATTG |  | This study |
| *B. fragilis wcfQ* gene |  | GTTTTGTGAAGCAGATCGCATTTAT  TCTTTTCCGTCATCATCAATTTCTAT |  | This study |
| *B. fragilis wcfQ* gene |  | TGCTATCATGGTTCGTGCTAGAA  TGAAAATTCCGACATGCATAATAAG |  | This study |
| *B. fragilis rplB* gene |  | TCAACCGGGTTCATAACAACAC  TAAGGCAGGTCGCTCTCGTT |  | This study |

**Table S2.** Fold diference (Log_2_) mRNA expression of ileal genes in mice treated with TCDD (30 μg/kg) or vehicle (sesame oil) by oral gavage once every four days for 28 days. Meaurements were taken using the nCounter*^®^* mouse immunology panel with mRNA expression of immunological genes in ileal tissue. Table only lists genes that were significantly influenced by TCDD, or colonization of SFB or *B. fragilis*. Comparisons were made between mice that remained uncolonized (UC), mice mono-colonized with *B. fragilis*, mice mono-colonized with SFB (SFB), and mice co-colonized with both bacteria (SFB+B).

| Gene | UC | | *B. fragilis* | | SFB | | SFB+*B. fragilis* | |
| --- | --- | --- | --- | --- | --- | --- | --- | --- |
|  | Veh | TCDD | Veh | TCDD | Veh | TCDD | Veh | TCDD |
| Abcf1 | - |  |  |  |  |  | -0.59 |  |
| B2m | - |  |  |  | 0.84 |  |  |  |
| Batf | - |  |  |  | 1.18 |  | 0.99 |  |
| Blnk | - | -1.26 |  |  |  |  |  |  |
| C4bp | - | -1.25 |  |  |  |  |  |  |
| Ccbp2 | - |  |  |  | 0.94 |  |  |  |
| Ccl5 | - |  |  |  | 2.16 |  | 1.96 |  |
| Ccl9 | - |  | 0.96 |  |  |  |  |  |
| Ccr2 | - |  |  |  | 1.45 |  |  |  |
| Ccr9 | - |  |  |  | 1.28 |  | 1.27 |  |
| Cd226 | - |  |  |  |  |  | 0.90 |  |
| Cd244 | - |  |  |  | 1.15 |  |  |  |
| Cd274 | - |  |  |  | 1.62 | -0.54 |  |  |
| Cd36 |  | 2.08 |  |  |  |  |  |  |
| Cd3d | - |  |  |  |  |  | 1.17 |  |
| Cd48 | - |  |  |  | 1.16 |  | 1.13 |  |
| Cd6 | - |  |  |  | 1.32 | -0.47 | 1.75 | -0.81 |
| Cd7 | - |  |  |  | 1.59 |  | 1.75 |  |
| Cd74 | - |  |  |  |  |  | 1.37 |  |
| Cd86 | - |  |  |  |  |  | 0.88 |  |
| Cfd |  |  |  |  |  |  |  | 1.68 |
| Cfi | - |  |  |  | 1.29 |  |  |  |
| Ciita | - |  |  |  | 1.90 | -0.71 | 1.53 | -0.53 |
| Ctnnb1 | - |  |  |  | 0.66 |  | 0.85 |  |
| Ctsc | - |  |  |  |  |  | 0.96 |  |
| Cxcr6 | - |  |  |  | 0.84 |  | 0.86 |  |
| Dpp4 | - |  |  |  | 0.83 |  | 0.70 |  |
| Fas | - |  |  |  | 1.48 |  | 1.10 |  |
| Fasl | - |  |  |  |  |  | 0.72 |  |
| Fcamr | - | -1.42 |  |  |  |  |  |  |
| Fkbp5 | - |  |  |  | -1.17 |  | -0.94 |  |
| Gpr44 | - |  |  |  |  |  | -1.11 |  |
| Gzma | - |  |  |  | 3.12 |  | 2.93 |  |
| Gzmb | - |  |  |  | 2.47 |  | 2.38 |  |
| H2-Aa | - |  |  |  | 2.43 |  | 2.42 | -0.53 |
| H2-Ab1 | - |  |  |  | 2.44 |  | 2.19 | -0.69 |
| H2-DMa | - |  |  |  | 1.39 |  | 1.52 |  |
| H2-Ea-ps | - |  |  |  | 1.21 | -0.95 |  |  |
| H2-Eb1 | - |  |  |  |  |  | 1.48 | -0.75 |
| H2-K1 | - |  |  |  |  |  | 0.71 |  |
| Hif1a | - |  |  |  |  |  | 0.90 |  |
| Icosl | - |  |  |  | 0.77 |  |  |  |
| Ikzf2 | - |  |  |  | 0.62 |  | 0.72 |  |
| Il12rb2 | - | 1.39 |  |  | 1.28 |  |  |  |
| Il13ra1 | - |  |  |  | 1.01 |  | 0.95 |  |
| Il15 | - |  |  |  | 1.06 |  |  |  |
| Il1b | - |  |  |  | 1.53 | -0.83 | 1.54 |  |
| Il1r2 |  |  |  |  |  | 0.78 |  |  |
| Il1rl1 | - |  |  |  | 0.74 |  |  |  |
| Il1rn |  |  |  |  |  | 1.34 |  |  |
| Il21 | - |  |  |  |  |  | 1.40 |  |
| Il2rb | - |  |  |  | 0.96 |  | 0.90 | -0.57 |
| Il33 | - |  |  |  | 1.27 |  | 1.23 |  |
| Il7 | - |  |  |  | 1.16 | -0.64 | 0.94 |  |
| Il7r | - |  |  |  | 0.71 | -0.48 | 0.59 |  |
| Irf1 | - |  |  |  | 0.78 |  | 0.77 |  |
| Itga4 | - |  |  |  |  |  | 1.24 |  |
| Itgb1 | - |  |  |  | 0.81 |  | 0.74 |  |
| Jak3 | - |  |  |  | 0.87 | -0.54 | 0.84 |  |
| Klrb1 | - |  |  |  | 1.63 |  |  |  |
| Klrd1 | - |  |  |  | 1.66 |  |  |  |
| Lck | - |  |  |  | 1.11 |  | 1.02 |  |
| Mme | - |  |  |  | 1.16 | -0.62 | 1.30 | -0.48 |
| Msr1 |  |  |  |  |  | 0.51 |  |  |
| Muc1 | - |  |  |  | 1.14 |  |  |  |
| Ncf4 |  |  |  |  |  | 0.90 |  |  |
| Nfil3 | - |  |  |  | 1.07 |  |  |  |
| Nfkbiz | - |  |  |  | 1.07 |  |  |  |
| Nos2 | - |  |  |  | 1.88 |  | 2.45 |  |
| Npc1 | - |  |  |  |  |  | 1.04 |  |
| Nt5e | - |  |  |  |  |  | 2.21 |  |
| Pdcd1 | - |  |  |  | 1.61 | -0.80 | 1.54 |  |
| Pdgfrb | - | -0.42 |  |  | -0.78 |  | -0.76 |  |
| Pigr | - |  |  |  | 1.12 |  | 1.36 |  |
| Pla2g2a | - |  |  |  | 1.23 |  |  |  |
| Plaur | - |  |  |  |  |  | 0.78 |  |
| Pparg | - |  |  |  |  | 0.92 |  | 1.33 |
| Prf1 | - |  |  |  |  |  | 1.42 |  |
| Psmb9 | - |  |  |  |  |  | 1.31 |  |
| Psmc2 | - |  |  |  | 1.17 |  | 0.94 |  |
| Psmd7 | - |  |  |  | 0.96 |  |  |  |
| Ptafr | - |  |  |  | 1.14 |  | 1.05 |  |
| Ptger4 | - |  |  |  |  |  | 0.85 |  |
| Ptk2 | - |  |  |  | 0.78 |  |  |  |
| Rorc | - |  |  |  | 0.71 |  |  |  |
| Serping1 | - |  |  |  | 0.99 |  |  |  |
| Sh2d1a | - |  |  |  |  |  | 1.51 |  |
| Socs3 | - | 0.63 |  |  |  |  | 1.42 |  |
| Stat2 | - |  |  |  |  |  |  |  |
| Stat3 | - |  |  |  | 0.82 |  | 0.78 |  |
| Tap1 | - |  |  |  |  |  | 0.78 |  |
| Tlr3 | - | -0.65 | -0.64 |  | -0.99 |  | -0.55 |  |
| Tlr8 | - |  |  |  | 1.45 |  | 1.22 |  |
| Tmem173 | - |  |  |  | 1.01 |  |  |  |
| Tnfrsf17 | - |  |  |  | 1.10 |  | 1.20 |  |
| Tnfrsf1b | - |  |  |  | 0.92 |  | 0.86 |  |
| Tnfsf13b | - |  |  |  |  | -0.27 | 1.08 |  |
| Tollip | - |  |  |  |  |  | 0.67 | -0.35 |
| Traf1 | - |  |  | -1.14 |  |  |  |  |
| Traf2 | - |  | 0.65 |  |  |  |  | -0.62 |
| Traf5 | - |  |  |  |  |  | 0.93 |  |
| Xbp1 | - |  |  |  | 0.69 |  | 0.63 |  |
| Zap70 | - |  |  |  | 1.20 |  |  |  |

**Table S3.** QPCR analysis showing average and standard error of SFB and *B. fragilis* expressed in the ileum and caecum, respectively, or copies (measued with DNA) from fecal pellets. “ND” indicates markers fell below the limit of detection.

| Group | Material isolated | Assay and matrix | Vehicle | TCDD |
| --- | --- | --- | --- | --- |
| SFB | RNA | SFB 16S rRNA genes expressed per mg iluem | 1.3 x 10^6^ + 6.4 x 10^5^ | 2.4 x 10^6^ + 1.0 x 10^6^ |
| SFB+B | RNA | SFB 16S rRNA genes expressed per mg iluem | 4.7 x 10^5^ + 1.7 x 10^5^ | 2.6 x 10^6^ + 1.4 x 10^6^ |
| SFB | DNA | SFB 16S rRNA gene copes per mg feces | 6.2 x 10^4^ + 2.0 x 10^4^ | 1.1 x 10^5^ + 1.3 x 10^4^ |
| SFB+B | DNA | SFB 16S rRNA gene copes per mg feces | 1.6 x 10^4^ + 6.6 x 10^3^ | 7 x 10^4^ + 2.6 x 10^4^ |
| SFB | DNA | SFB flic gene copies per mg feces | 8.0 x 10^3^ + 2.5 x 10^3^ | 1.3 x 10^4^ + 1.4 x 10^3^ |
| SFB +B | DNA | SFB flic gene copies per mg feces | ND | ND |
| SFB | RNA | SFB putative rubrerythrin gene relative expression in ileum | 1.6 x 10^-4^ + 4.8 x 10^-5^ | 9.0 x 10^-5^ + 4.1 x 10^-6^ |
| SFB+B | RNA | SFB putative rubrerythrin gene relative expression in ileum | ND | ND |
| B | RNA | *B. fragilis* specific *rplB* genes expressed per mg caecum | 9.2 x 10^2^ + 1.8 x 10^2^ | 2.9 x 10^2^ + 4.7 x 10^1^ |
| SFB+B | RNA | *B. fragilis* specific *rplB* genes expressed per mg caecum | 1.3 x 10^3^ + 3.1 x 10^2^ | 7.8 x 10^2^ + 2.2 x 10^1^ |


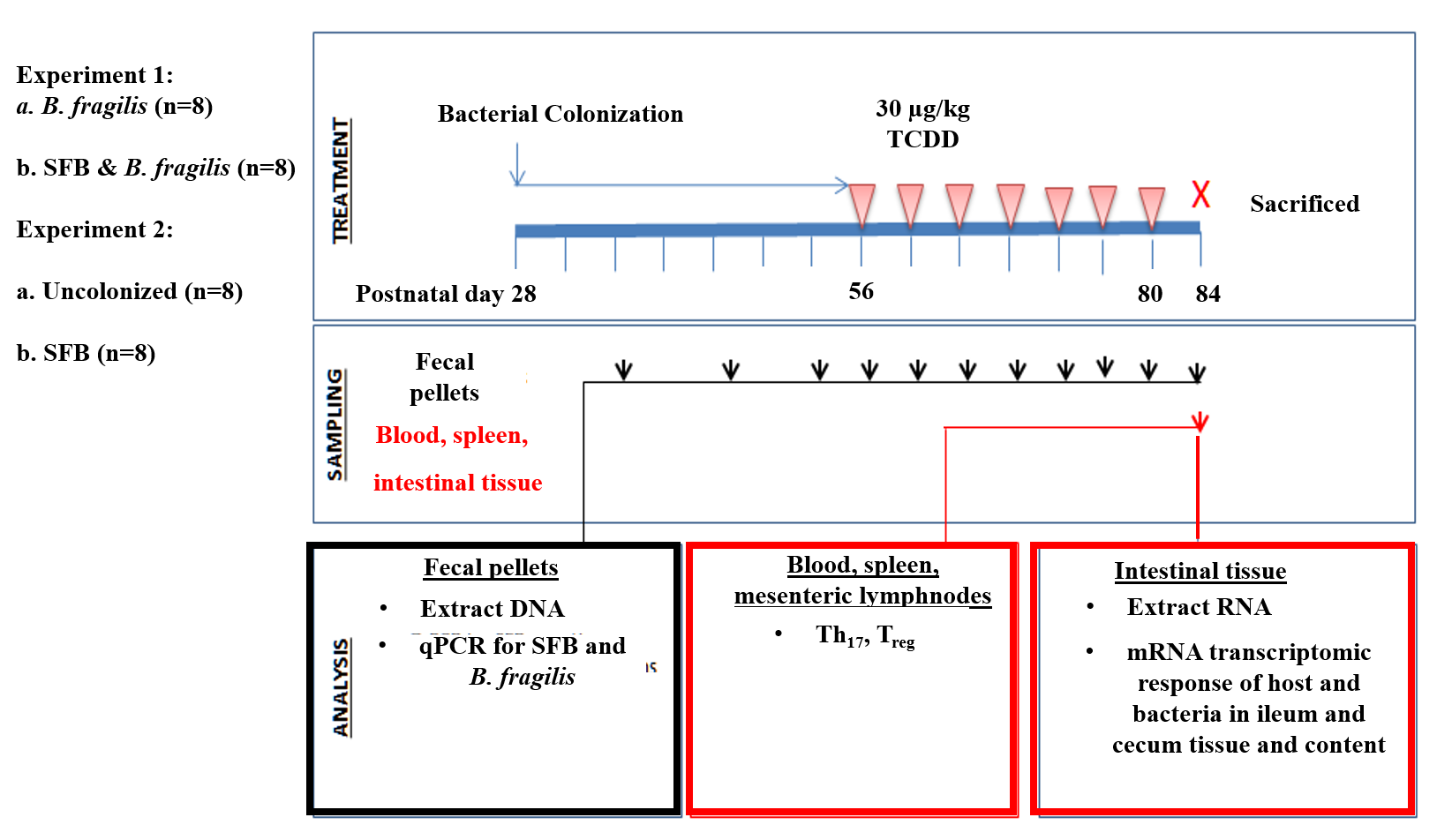
Fig S1. Details of experimental design. Female C57BL/6 gnotobiotic mice were orally gavaged with vehicle of sesame oil or 30 µg/kg TCDD every 4 days for 28 days. Treatment of TCDD started 28 days post colonization of bacteria with SFB, *B. fragilis*, both SFB and *B. fragilis* (SFB+B), or remained uncolonized. Fecal pellets were collected throughout the study to ensure proper bacterial colonization. Blood, spleen, and mesenteric lymphnodes and gut tissues were collected post-sacrifice.

**
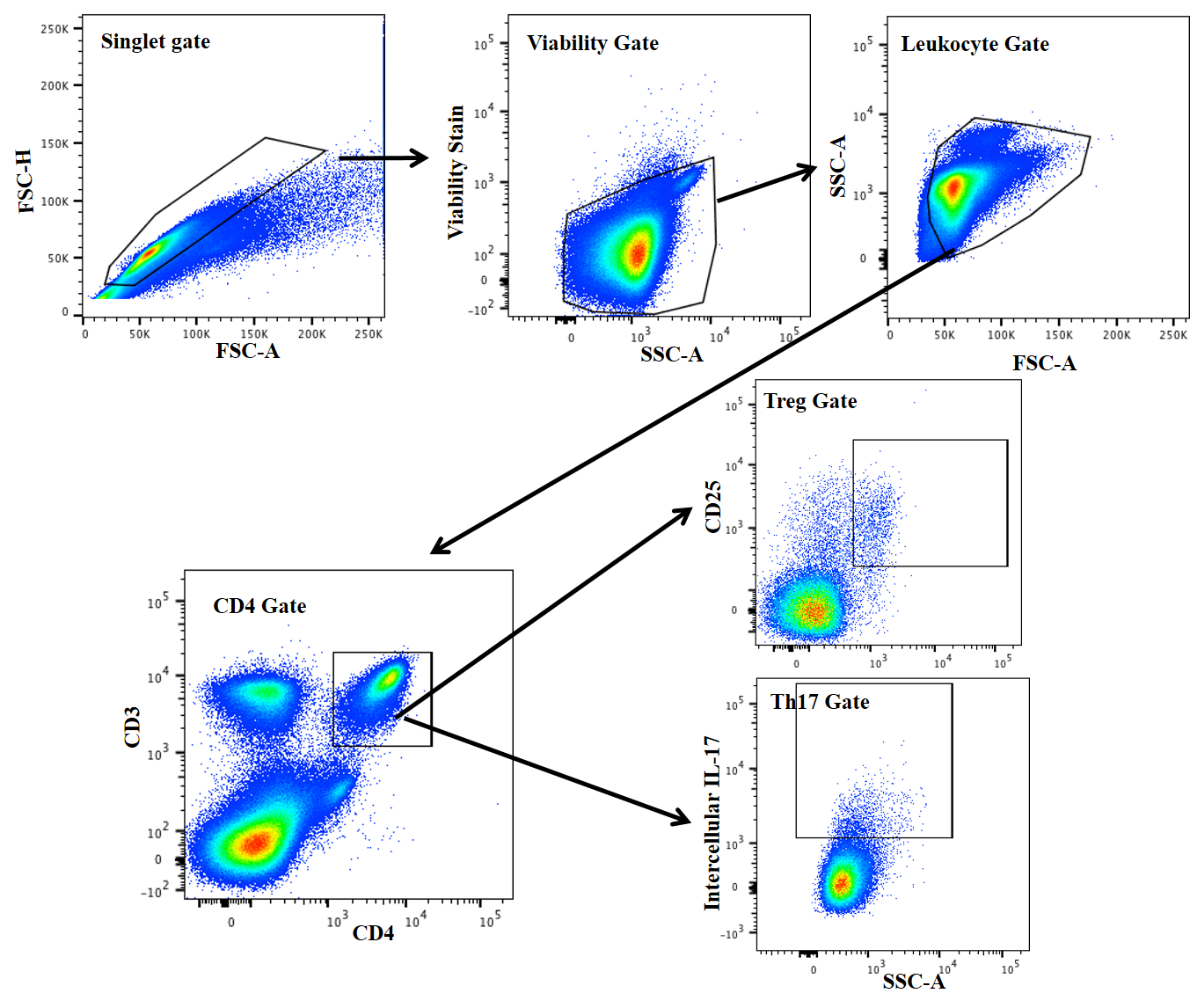
Fig S2.** Gating strategy for identifying the T_reg_ and Th_17_ cell populations. This figure above represents the gating strategy used for identification and quantification of the Treg (CD4+, CD25+ and FoxP3+) and Th17 (CD4+ and intercellular IL-17+) populations. This gating strategy was employed for all of the tissues analyzed (mesenteric lymph nodes, spleen and trunk blood). This example shows analysis of trunk blood collected from the vehicle dosed mouse colonized with *B. fragilis* and SFB. Red blood cells were lysed and the remaining leukocytes were stained as previously described.

**
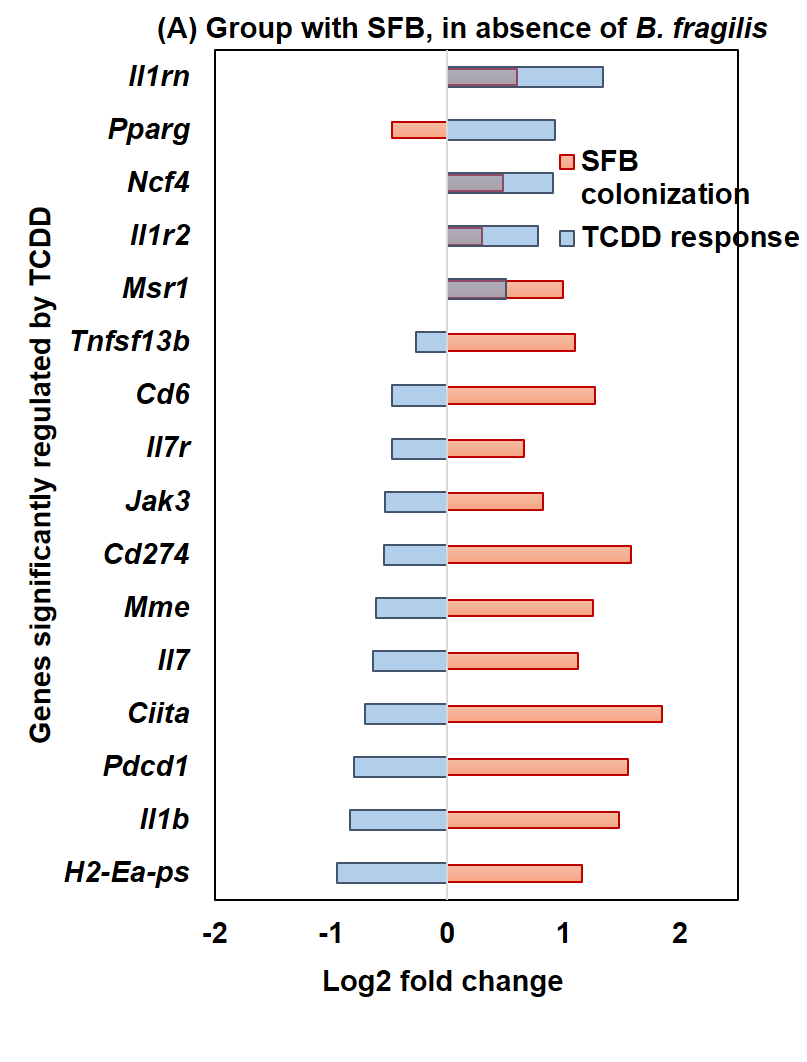
**

**Fig S3.** Verifying opposing response of TCDD and SFB in mice mono-colonized with SFB (no *B. fragilis*). nCounter*^®^* mouse immunology panel analysis showing all genes in ileal tissue that were significantly influenced in response to TCDD in mice solely colonized with SFB. Bar color indicate up/down regulation due to TCDD (blue). For genes influenced by TCDD, fold change expression due to SFB colonization is shown regardless of significance. For comparative analysis of TCDD and SFB colonization (red), the vehicle dosed uncolonized group was compared with the group mono-colonized with SFB.


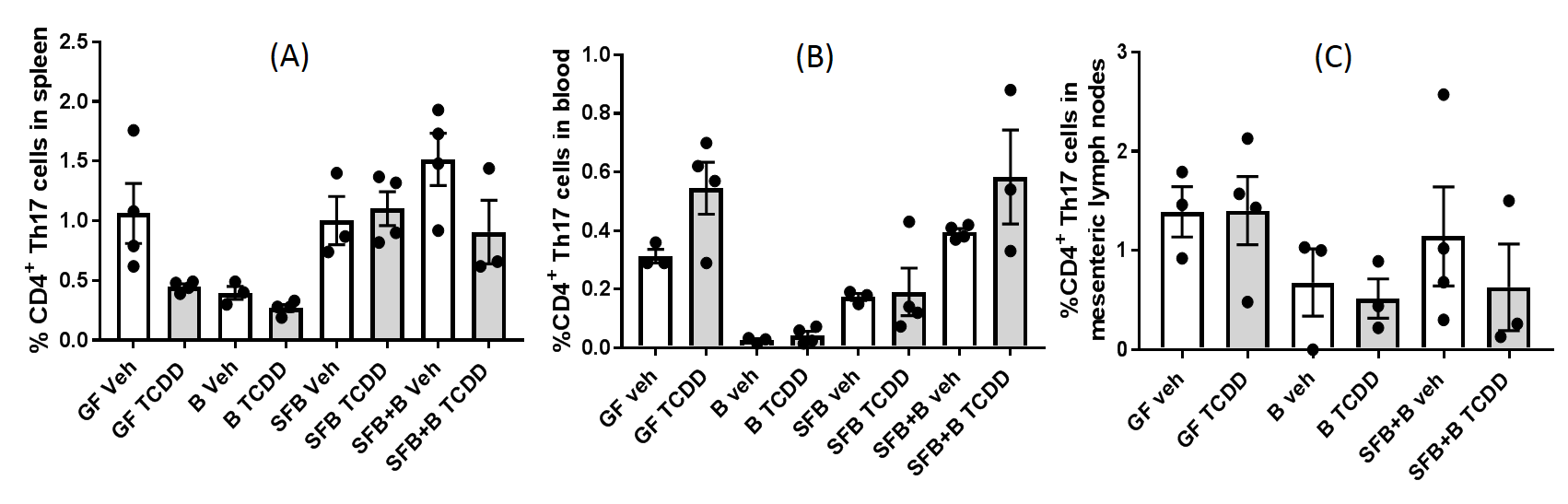
**Fig S4.** Percent CD4^+^ Th_17_ measured in (A) whole blood, (B) spleen, and (C) mesenteric lymph nodes in C57BL/6 female mice after TCDD (30 μg/kg) or vehicle (sesame oil) treatment by oral gavage once every four days for 28 days. Gray bars are TCDD dosed and white bars are vehicle dosed mice. Values represent mean percent and error bars represent standard error. Abbreviations include mice that remained uncolonized (GF), mono-colonized with SFB (SFB), mono-colonized with *B. fragilis* (B) and co-colonized (SFB+B). Th_17_ cells were not measured in mesenteric lymph node of mice mono-colonized with SFB.

**
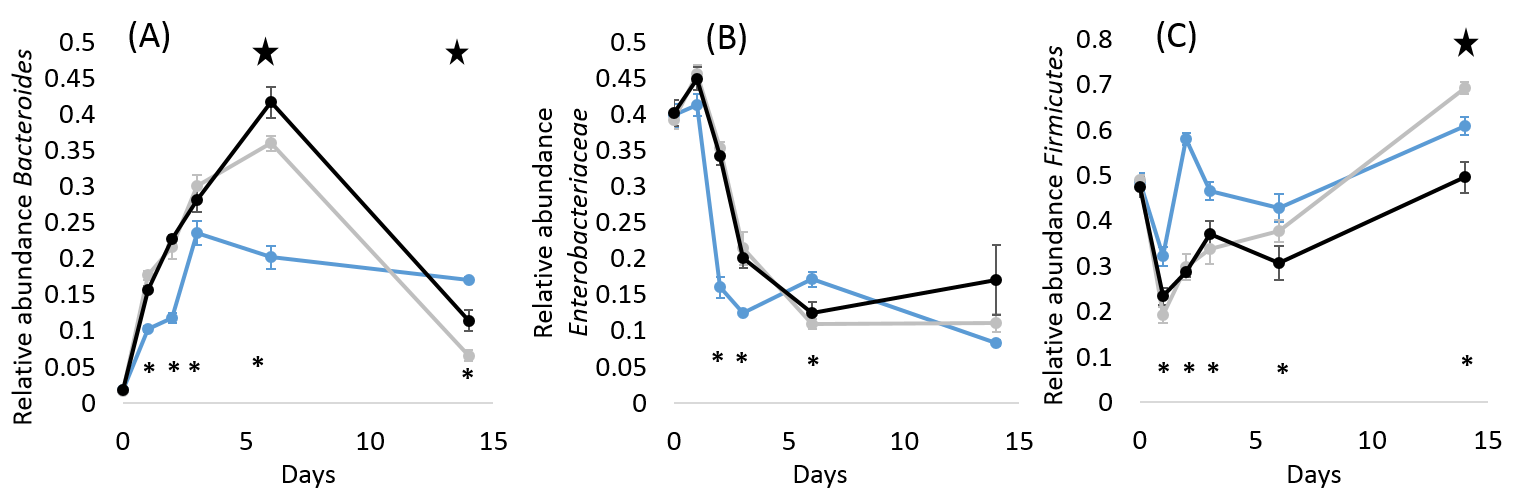
Fig S5.** 16S rRNA gene analysis of TCDD influence on commensals in-vitro. Mean relative abundance based on taxonomic classification of (A) *Bacteroides*, (B) *Enterobacteriaceae*, which responds similarly to SFB in mice with a traditional gut microbiome, and (C) *Firmicutes* based on 16S rRNA gene V4 region. Samples were collected on day 0, 1, 2, 3, 6, 14 days after addition of TCDD and sesame oil. Blue, gray, black lines indicate naïve, vehicle of sesame oil, and 30 nmol/L TCDD in vehicle, respectively. Error bars indicate standard error between five microcosms. Stars indicate significant difference (p < 0.05) between sesame oil alone and sesame oil with TCDD. Points marked with “*” indicate significant difference (p < 0.05) between naïve and sample with sesame oil. Notice a significant difference in *Bacteroides* and *Firmicutes* on day 6 and 14; however, shifts are opposite of TCDD induced indirect response observed in mice.

**Supplemental References**

Barman, M., Unold, D., Shifley, K., Amir, E., Hung, K., Bos, N., et al. (2008). Enteric salmonellosis disrupts the microbial ecology of the murine gastrointestinal tract. *Infect Immun* 76, 907–15.

Bouskra, D., Brézillon, C., Bérard, M., Werts, C., Varona, R., Boneca, I. G., et al. (2008). Lymphoid tissue genesis induced by commensals through NOD1 regulates intestinal homeostasis. *Nature* 456, 507–10. doi:10.1038/nature07450.

Guo, X., Xia, X., Tang, R., Zhou, J., Zhao, H., and Wang, K. (2008). Development of a real-time PCR method for Firmicutes and Bacteroidetes in faeces and its application to quantify intestinal population of obese and lean pigs. *Lett Appl Microbiol* 47, 367–73.

Kozich, J., Westcott, S., Baxter, N., Highlander, S., and Schloss, P. (2013). Development of a dual-index sequencing strategy and curation pipeline for analyzing amplicon sequence data on the MiSeq Illumina sequencing platform. *Appl Env. Microbiol* 79, 5112–20.

Lane, D. J. (1991). *rRNA sequencing*. , eds. M. Goodfellow and E. Stackebrandt J. Wiley.

Larsen, N., Vogensen, F. K., van den Berg, F. W. J., Nielsen, D. S., Andreasen, A. S., Pedersen, B. K., et al. (2010). Gut microbiota in human adults with type 2 diabetes differs from non-diabetic adults. *PLoS One* 5, e9085.

Lay, C., Sutren, M., Rochet, V., Saunier, K., Doré, J., and Rigottier-Gois, L. (2005). Design and validation of 16S rRNA probes to enumerate members of the *Clostridium leptum* subgroup in human faecal microbiota. *Env. Microbiol* 7, 933–946.

Layton, A., McKay, L., Williams, D., Garrett, V., Gentry, R., and Sayler, G. (2006). Development of *Bacteroides* 16S rRNA gene TaqMan-based real-time PCR assays for estimation of total, human, and bovine fecal pollution in water. *Appl Env. Microbiol* 72, 4214–4224.

Matsuki, T., Watanabe, K., Fujimoto, J., Miyamoto, Y., Takada, T., Matsumoto, K., et al. (2002). Development of 16S rRNA-gene-targeted group-specific primers for the detection and identification of predominant bacteria in human feces. *Appl Env. Microbiol* 68, 5445–51.

Prakash, T., Oshima, K., Morita, H., Fukuda, S., Imaoka, A., Kumar, N., et al. (2011). Complete genome sequences of rat and mouse segmented filamentous bacteria, a potent inducer of th17 cell differentiation. *Cell Host Microbe* 10, 273–84. doi:10.1016/j.chom.2011.08.007.

Ramirez-Farias, C., Slezak, K., Fuller, Z., Duncan, A., Holtrop, G., and Louis, P. (2009). Effect of inulin on the human gut microbiota: stimulation of *Bifidobacterium adolescentis* and *Faecalibacterium prausnitzii.* *Br J Nutr* 101, 541–550.

Rinttilä, T., Kassinen, A., Malinen, E., Krogius, L., and Palva, A. (2004). Development of an extensive set of 16S rDNA-targeted primers for quantification of pathogenic and indigenous bacteria in faecal samples by real-time PCR. *J Appl Microbiol* 97, 1166–1177.

Robinson, C. D., Auchtung, J. M., Collins, J., and Britton, R. A. (2014). Epidemic *Clostridium difficile* strains demonstrate increased competitive fitness compared to nonepidemic isolates. *Infect Immun* 82, 2815–25.

Sghir, A., Gramet, G., Suau, A., Rochet, V., Pochart, P., and Dore, J. (2000). Quantification of bacterial groups within human fecal flora by oligonucleotide probe hybridization. *Appl Env. Microbiol* 66, 2263–6.

Stevenson, M, D., and Weimer, P. J. (2007). Dominance of Prevotella and low abundance of classical ruminal bacterial species in the bovine rumen revealed by relative quantification real-time PCR. *Appl Microbiol Biotech* 75, 165–74.

Van Dyke, M. I., and McCarthy, A. J. (2002). Molecular biological detection and characterization of Clostridium populations in municipal landfill sites. *Appl Env. Microbiol* 68, 2049–2053.

Vital, M., Penton, C. R., Wang, Q., Young, V. B., Antonopoulos, D. A., Sogin, M. L., et al. (2013). A gene-targeted approach to investigate the intestinal butyrate-producing bacterialcommunity. *Microbiome* 1, 1.

Walker, A. W., Duncan, S. H., Leitch, E. C. M., Child, M. W., and Flint, H. J. (2005). pH and peptide supply can radically alter bacterial populations and short-chain fatty acid ratios within microbial communities from the human colon. *Appl Env. Microbiol* 71, 3692–3700.

Wang, R.-F., Cao, W.-W., and Cerniglia, C. E. (1997). PCR detection of *Ruminococcus* spp. in human and animal faecal samples. *Mol Cell Probes* 11, 259–265.
